# Social media-related nightmare — a potential explanation for poor sleep quality and low affective well-being in the social media era?

**DOI:** 10.1186/s40359-024-01605-z

**Published:** 2024-03-12

**Authors:** Reza Shabahang, Sohee Kim, Mara S. Aruguete, Pegah Azadimanesh, Zahra Ghaemi, Abbas Ali Hossein Khanzadeh, Keivan Kakabaraee, Ágnes Zsila

**Affiliations:** 1 Department of Psychology, College of Education, Psychology and Social Work, Flinders University, Adelaide, Australia; 2 Department of Counseling and Instructional Sciences, University of South Alabama, Mobile, AL USA; 3 Department of Social and Behavioral Sciences, Lincoln University, Missouri, MO USA; 4 Department of Psychology, University of Guilan, Rasht, Iran; 5 Department of Psychology, Kermanshah Branch, Islamic Azad University, Kermanshah, Iran; 6 Institute of Psychology, Pázmány Péter Catholic University, Budapest, Hungary; 7 Institute of Psychology, ELTE Eötvös Loránd University, Budapest, Hungary

**Keywords:** Anxiety, Dreaming, Nightmares, Sleep quality, Social media use

## Abstract

Research has posited that social media use during the day may be reflected in nighttime dreams. Nevertheless, no prior studies have explored frightening, unpleasant dreams arising from social media use. This study introduces the construct of the *social media-related nightmare* by (a) developing and validating a scale capturing negative-valenced dreams with themes of helplessness, loss of control, inhibition, victimization, and making mistakes in social media, and (b) examining relationships between social media use, social media-related nightmares, sleep quality, and affective well-being. A convenience sample of 595 Iranian adult social media users (*M*_*age*_ = 27.45, *SD*_*age*_ = 11.42) reported on social media-related nightmare, social media use integration, anxiety, peace of mind, sleep quality, and nightmare distress. The *Social Media-Related Nightmare Scale* (*SMNS*) demonstrated a unidimensional structure with sound psychometric properties. The most common nightmares involved the inability to log in to social media and the disruption of relationships with other users. Social media use intensity predicted frequency of social media-related nightmares. These nightmares were correlated with increased anxiety, lower peace of mind, poor sleep quality, and nightmare distress. Importantly, social media-related nightmares mediated the relationship between social media use intensity and low affective well-being (i.e., anxiety and peace of mind), poor sleeping, and nightmare distress. The findings suggest that social media-related nightmares could be a potential pathway through which social media engagement may lead to affective distress and sleep difficulties.

## Introduction

The use of social media has rapidly increased in recent years, with a majority of users checking social media platforms daily, placing them at risk of social media addiction [1]. This surge in social media popularity has led to a growing interest in the potential impact of daytime media engagement on nighttime dream content. In line with the *Continuity Hypothesis of Dreaming*, positing that walking-life experiences are reflected in dreams [2], a few studies have explored the influence of media use, including social media, on dream content. Interestingly, findings have shown a significant association between media use, particularly social media use, and the frequency of media-related dreams [3, 4]. Notably, the age of initial social media use and frequency of use have been identified as contributing factors to dream intensity [5].

While studies have examined the association of social media engagement with generic dreaming [5] and social media-related specific dreams [4], scant attention has been paid to the contribution of social media usage to negative-valenced dreams (nightmares) with content centering around social media. Despite the low incidence of social media dreams [4], these dreams have been found to be associated with negative psychological states (e.g., neuroticism; [4]), emphasizing the importance of examining this type of dream. With increasing integration of social media use (time spent) in the community and users’ growing concerns associated with social media use (e.g., identity theft, fake online identities, information leaks, misinformation, and online harassments), dreams with content related to social media are expected to gradually increase. Nightmares are intertwined with psychological problems, such as depression and anxiety, and can serve as risk factors for subsequent anxiety disorders and suicidal thoughts [6].

To date, research has explored various nightmare themes, many of which revolve around social concerns. For instance, Schredl [7] identified five primary nightmare themes: falling, being chased, being paralyzed, being late, and having close persons disappear or die. Robert and Zadra [8] classified nightmares into 12 themes, encompassing physical aggression, interpersonal conflicts, failure or helplessness, health-related concerns and death, apprehension, being chased, evil force, accident, disaster and calamity, insects, and environmental abnormality. Schredl et al. [9] added themes of death/injury to others, falling, being lucid, examination, sexual aggression, being the aggressor, and suicide. Furthermore, helplessness, loss of control, inhibition, victimization, and making mistakes are among other established nightmare themes [10]. However, what remains unclear is the presence of nightmares centered around social media-rooted distress.

Social media users harbor risk beliefs and concerns stemming from social media use [11], such as apprehension of vulnerability in social media [12], fear of rejection, and a desire for social approval [13]. The *Neurocognitive Model of Nightmare Etiology* [14] posits that *affect load* (i.e., frequency of current life stress and/or negative emotional events that impede emotion processing capacities) and *affect distress* (i.e., dispositional tendency to experience heightened negative emotional reactivity) contribute to the pathogenesis of nightmares. Given that concerns over social media engagement are expected, these concerns may manifest in nightmares [15]. Inspired by existing studies on nightmares and their themes, social media-related nightmares can be defined as negative-valenced dreams about social media, primarily characterized by themes of helplessness, loss of control, inhibition, victimization, and making mistakes. To distinguish social media-related nightmares, which are disturbing dreams awakening the sleeper, from social media-related bad dreams [16], the awakening of the sleeper needs to be considered a key attribute for social media-related nightmares.

Research on the interplay between social media use, sleep, and dreams is still in its early stages. There are unanswered questions about how users’ sleep patterns and dream contents are influenced by social media. To date, a plethora of research has demonstrated that excessive social media use is associated with poor sleep quality, contributing to mental and physical health problems [17]. Findings have suggested that problematic social media use affects sleep both directly and indirectly, leading to poor sleep quality and negative affect. These consequences of excessive social media use may help explain the observed low life satisfaction in problematic social media users [18]. Moreover, excessive social media use appears to be related to an increase in nightmares across genders and countries [19]. Social media engagement has been reported to be linked to problematic dreaming, greater aggression in dreams, dream intensity, and negatively valenced dreams [3, 5, 19, 20].

Numerous studies have also highlighted the association between intensity of social media use and depression, anxiety, and psychological distress [21]. The extensive integration of social media into daily life can lead to worry, depressive rumination, and co-rumination [22, 23], with the potential to deteriorate users’ peace of mind [24].

Although abundant investigations have traced the link between social media use, sleep, dreams, and mental health, there is still room to further examine these associations. No studies have yet shed light on nightmares shaped by today’s social media. Guided by the continuity hypothesis of dreaming [2], the neurocognitive model of nightmare etiology [14], and previous research on nightmare content [7–10], this study aimed to introduce and quantify social media-related nightmares by constructing and validating a self-report assessment. Furthermore, the current investigation attempted to examine the association between social media use integration with sleep (i.e., sleep quality and nightmare distress) and mental health (i.e., anxiety and peace of mind) by exploring the mediating role of social media-related nightmares.

## Methods

### Participants

Participants were 595 Iranian adult social media users (*M*_*age*_ = 27.45, *SD*_*age*_ = 11.42; 405 women and 190 men) who completed an online survey in February 2022. To qualify for participation, respondents must have been users of at least one social media platform, with a minimum usage of 15 min of social media per day during the last month. Most of the participants indicated Instagram as the social media platform they used most often (87% Instagram, 11% Twitter, and 2% Facebook).Ethical practices (e.g., maintaining confidentiality and anonymity) were observed in accordance with the World Medical Association Declaration of Helsinki and the American Psychological Association. Participation was voluntary and online informed consent was mandatory prior to participation. This study was approved by the Institutional Review Board of the University of Guilan.

### Measures

For this study, the *Social Media Nightmare-Related Scale* (*SMNS*) was developed, consisting of 14items (see Table 1). The scale construction drew upon previous classifications of nightmares, literature discussing nightmares [7–9], studies exploring the influence of media on dreaming [3, 4], and common concerns expressed by social media users [11–13]. First, we synthesized literature on the content of dreaming [2, 10], nightmare categories [7–9], and the interrelationship between media and dreams [4, 5] to create a cohesive summary, establishing a robust framework for capturing social media-related nightmares. Initial items were formulated drawing on established measures assessing dream content, such as the Typical Dream Questionnaire (e.g., being chased or pursued, trying repeatedly to accomplish something, and being tied or unable to move; [10]), and proposed nightmare categories, such as Robert and Zadra’s [8] thematic categories in nightmares and bad dreams (e.g., being chased, interpersonal conflicts, accidents, failures, and helplessness; [8]). To enrich the connotation of the items, common concerns of social media users were also considered, such as harassment, data breach, posting anxiety, and social disapproval [11–13]. Subsequently, the initial items underwent refinement based on feedback from three media psychologists with expertise in scale development. Their comments and revisions focused on enhancing the clarity, simplicity, and relevance of the items [25].

Participants were asked to indicate the frequency with which they experienced nightmares related to these social media specific topics, such as “Disruption of relationships with other social media users”. Participants were instructed to specifically focus on nightmares– those distressing dreams that awaken them from sleep–with the aim of capturing nightmares rather than general bad dreams. The items were crafted to center around themes of helplessness, loss of control, inhibition, victimization, and making mistakes within the realm of social media. Response options ranged from 0 (*never*) to 7 (*several times a week*).

**Table 1:** Items of the *Social Media-Related Nightmare Scale*

How often do you experience nightmares about the following topics?
1. Receiving threat messages in social media
2. Being insulted and humiliated in social media
3. Being unable to log in to social media
4. Being rejected by another social media user
5. Disruption of relationships with other social media users
6. Being isolated in social media without other social media users
7. Being unable to communicate in social media despite the presence of other social media users
8. Being anonymous in social media (without identity)
9. Being lost in social media
10. Someone stealing your identity in social media
11. Being sexually harassed in social media
12. Posting an inappropriate post on social media (intentionally or unintentionally)
13. Writing an inappropriate comment for a post (intentionally or unintentionally)
14. Sharing your personal information on social media (intentionally or unintentionally)

The 10-item *Social Media Use Integration Scale* (*SMUIS*; [26]) assesses the intensity of social media use by gauging the extent to which it is integrated into daily life. The scale comprises two subscales: Social Integration and Emotional Connection (6 items, e.g., “I prefer to communicate with others mainly through social media platforms”) and Integration into Social Routines (4 items, e.g., “Using social media is part of my everyday routine”). Respondents use Likert-type response options (1 = *strongly disagree*, 6 = *strongly agree*). The scale has demonstrated satisfactory validity and reliability. The SMUIS and its subscales were internally consistent (*α*_*Social Integration and Emotional Connection Subscale*_ = 0.85; *α*_*Integration into Social Routines Subscale*_ = 0.69; *α*_*Social Media Use Integration*_ = 0.87) in the present study.

The 6-item *Anxiety Subscale* of the *Brief Symptom Inventory* (*BSI*; [27]) assesses the degree to which anxiety symptoms, such as “Nervousness or shakiness inside”, have bothered respondents in the past seven days. Respondents rate each item on a 5-point Likert scale (0 = *not at all*, 4 = *extremely*). In the present study, the anxiety subscale demonstrated excellent reliability with an *α* coefficient of 0.91.

The 7-item *Peace of Mind Scale* (*PoM*; [24]) assesses internal peace and ease in daily life, with items such as “My mind is free and at ease”. This unidimensional scale utilizes a 5-point Likert scale (1 = *not at all*, 5 = *all of the time*), including two reverse-scored items. The internal consistency of the PoM in the current study was sound, with a reliability coefficient (*α*) of 0.90.

The single-item *Sleep Quality Scale* (*SQS*; “During the past 7 days, how would you rate your sleep quality overall?” [28]) evaluates the overall quality of sleep over a 7-day recall period. Respondents are asked to rate their sleep quality on a discretizing visual analog scale ranging from 0 (*terrible*) to 10 (*excellent*) by considering various aspects such as sleep duration, ease of falling asleep, and waking up during sleep.

The 13-item *Nightmare Distress Questionnaire* (*NDQ*; [29]) assesses subjective feelings following nightmares and comprises three subscales: the General Nightmare Distress Subscale (e.g., “Do you have difficulties coping with nightmares?”), the Impact on Sleep Subscale (e.g., “Are you ever afraid to fall asleep for fear of having a nightmare?”), and the Impact on Daily Reality Perception Subscale (e.g., “Do you ever find yourself avoiding or disliking or fearing someone because they were in your nightmare?”). Respondents use Likert-type response options, with 10 items rated from 1 (*never*) to 5 (*always*), 2 items from 1 (*not at all*) to 5 (*a great deal*), and 1 item from 1 (*not at all interested*) to 5 (*extremely interested*) [30]. Internal consistency of the NDQ and its subscales demonstrated adequacy in the present study (*α*_*General Nightmare Distress Subscale*_ = 0.76; *α*_*Impact on Sleep Subscale*_ = 0.79; *α*_*Impact on Daily Reality Perception Subscale*_ = 0.76; *α*_*Nightmare Distress*_ = 0.91).

### Procedure and statistical analysis

An online survey, incorporating the mentioned measures along with demographic factors (age and gender), was created and administered using Google Forms. The survey link was distributed by posting on two prominent online shopping sites in Iran. Psychometric validation of the Social Media Nightmare-Related Scale was conducted through factor analysis (Exploratory Factor Analysis [EFA] and Confirmatory Factor Analysis [CFA]) and item analysis (corrected item-total correlations, Cronbach’s alpha, McDonald’s omega value, and Spearman-Brown split-half reliability). Demographic variations related to age and gender in social media-related nightmares were examined using Analysis of Variance (ANOVA). Second-order Structural Equation Modeling (SEM) was employed to elucidate the associations between the study’s variables.

Model fit for factor and structural analyses was evaluated using the Comparative Fit Index (CFI; ≥ 0.95 for excellent, ≥ 0.90 for good), the Tucker-Lewis Index (TLI; ≥ 0.95 for excellent, ≥ 0.90 for good), and the root-mean-square error of approximation (RMSEA; ≤ 0.06 for excellent, ≤ 0.08 for good), considering established cutoffs [31, 32]. Additionally, cutoff points of ≥ 0.80 for McDonald’s omega value [33] and ≥ 0.70 for Spearman-Brown split-half reliability [34] were considered. Based on the suggestion by Reckase [35], a set of items for the EFA could be considered as unidimensional if the first factor accounts for 20% or more of the total variance. Data analyses were performed using IBM SPSS Statistics Package for Windows, version 25.0 [36], and the lavaan package [37] in R software [38].

## Results

### Psychometric properties of the social media-related nightmare scale (SMNS)

To validate the Social Media-Related Nightmare Scale (SMNS), the sample (*N* = 595) was randomly split into two halves. The first half (*n* = 290) was utilized to explore the potential number of underlying factors through Exploratory Factor Analysis (EFA), and the detected factors were cross-validated in the second half (*n* = 305) using Confirmatory Factor Analysis (CFA). The randomly split samples for EFA and CFA were equivalent, and no significant differences in age and gender diversity were observed between the two sub-samples.

Factor analysis was conducted using principal component analysis (PCA) without rotation. The Kaiser-Meyer-Olkin (KMO) measure yielded a value of 0.87, and Bartlett’s test of sphericity was significant (*χ2*(91) = 1235.37,*p* <.001), indicating satisfactory sampling adequacy and factorability of the correlation matrix, respectively. Analysis of the scree plot and component matrix output revealed a one-factor solution comprising 14 items (eigenvalue = 5.12), with robust factor loadings (see Table 2). This first component explained 36.6% of the variance, confirming that the item set could be considered unidimensional.

Then, CFA was conducted with the remaining randomly split data (*n* = 305) to cross-validate the factor structure. Assessing the fit indices against established cutoff points, the one-dimensional CFA model demonstrated values indicative of a good fit (*χ2*(66) = 116.412, *p* <.001; CFI = 0.941; TLI = 0.918; RMSEA = 0.050, 90% CI: 0.035– 0.065; and SRMR = 0.046). Factor loadings ranged from 0.270 to 0.700.

Subsequently, the item characteristics of the SMNS were assessed using the full sample of 595 responses. A Cronbach’s alpha of 0.83 was obtained, indicating strong internal consistency. The corrected item-total correlations were excellent, ranging from 0.37 to 0.59. Additionally, the McDonald’s omega value (*ω* = 0.83; cutoff of ≥ 0.80) and the Spearman-Brown split-half reliability (*r*_*Spearman−Brown*_ = 0.77; cutoff of ≥ 0.70) surpassed the recommended cutoff benchmarks, confirming the high internal consistency of the scale (see Table 2).

**Table 2:** Item characteristics (mean and standard deviation), communality, and factor loadings from EFA of the Social Media-Related Nightmare Scale (SMNS)

	M*	SD*	Corrected Item-Total Correlation*	${\varvec{h}}^{2}$**	Factor loading**
*1*	0.261	0.667	0.433	0.579	0.630
*2*	0.355	0.761	0.560	0.620	0.713
*3*	0.610	1.011	0.463	0.403	0.536
*4*	0.390	0.763	0.567	0.492	0.686
*5*	0.568	0.888	0.590	0.553	0.691
*6*	0.350	0.850	0.495	0.490	0.559
*7*	0.444	0.935	0.513	0.527	0.620
*8*	0.466	1.060	0.431	0.668	0.578
*9*	0.259	0.701	0.480	0.493	0.608
*10*	0.155	0.475	0.412	0.571	0.619
*11*	0.104	0.366	0.365	0.522	0.530
*12*	0.215	0.617	0.365	0.492	0.541
*13*	0.229	0.599	0.390	0.521	0.523
*14*	0.203	0.536	0.437	0.530	0.591

Note. *N = 595, **n = 290;$$ {h}^{2}$$= communality from EFA; Factor loading = Factor loading from EFA.

### Age, gender, and social media-related nightmare

A two-way ANOVA was conducted (completely randomized factorial design) to examine whether social media-related nightmare frequency varies by gender and age group. The average score of fourteen items in the SMNS served as the dependent variable, while gender and age group variables were employed as independent variables for the ANOVA. The age group was categorized by two levels: under 27 or over 27, since the average age was 27.75. The two-way ANOVA results revealed no significant interaction effect between gender and age (*F*_1,591_ = 2.226, *p* >.05). In light of the non-significant interaction, we proceeded to investigate the main effect of each independent variable. However, there were no significant main effects observed for gender and age (gender: *F*_1,591_ = 0.562, *p* >.05; age group: *F*_1,591_= 0.373, *p* >.05). Consequently, no substantial differences in social media-related nightmare frequency were identified across gender and age groups.

### Content of social media-related nightmares

Overall, respondents reported a low prevalence of social media-related nightmares (*M* = 0.33, *SD* = 0.42). According to the descriptive statistics for each item in the SMNS (see Table 3), “Being unable to log in to social media (item 3)” was the most common nightmare (*M* = 0.61, *SD* = 1.01) and “Disruption of relationships with other social media users (item 5)” was the second most common nightmare (*M* = 0.57, *SD* = 0.89). Whereas “Being sexually harassed in social media (item 11)” was the least common nightmare (*M* = 0.10, *SD* = 0.37) and “Someone stealing your identity in social media (item 10)” was the second least common nightmare (*M* = 0.15, *SD* = 0.47).

**Table 3:** Item characteristics (mean and standard deviation) of the Social Media-Related Nightmare Scale (SMNS)

	Items	M	SD
*1*	Receiving threat messages in social media	0.26	0.67
*2*	Being insulted and humiliated in social media	0.35	0.76
*3*	Being unable to log in to social media	0.61	1.01
*4*	Being rejected by another social media user	0.39	0.76
*5*	Disruption of relationships with other social media users	0.57	0.89
*6*	Being isolated in social media without other social media users	0.35	0.85
*7*	Being unable to communicate in social media despite the presence of other social media users	0.44	0.93
*8*	Being anonymous in social media (without identity)	0.47	1.06
*9*	Being lost in social media	0.26	0.70
*10*	Someone stealing your identity in social media	0.15	0.47
*11*	Being sexually harassed in social media	0.10	0.37
*12*	Posting an inappropriate post on social media (intentionally or unintentionally)	0.22	0.62
*13*	Writing an inappropriate comment for a post (intentionally or unintentionally)	0.23	0.60
*14*	Sharing your personal information on social media (intentionally or unintentionally)	0.20	0.54

*Note. N* = 595.

#### Mediating role of social media-related nightmares

Before conducting CFA and SEM analysis with the aim of investigating the association between study’s variables and determining the mediating effect of social media-related nightmares, correlations among the measures were investigated in order to have a preliminary overview on the interconnections (see Table 4). Social media-related nightmare showed a positive correlation with social media use integration (*r* =.21, *p* <.001), anxiety (*r* =.31, *p* <.001), and nightmare distress (*r* =.31, *p* <.001). Peace of mind (*r* = −.26, *p* <.001) and sleep quality (*r* = −.21, *p* <.001) were negatively correlated to social media-related nightmare.

**Table 4:** Correlations among the variables in the study

	1	2	3	4	5	6
**1. Social media-related nightmare**	1	0.21***	0.31***	− 0.26***	− 0.21***	0.31***
**2. Social media use integration**		1	0.28***	− 0.13**	− 0.10*	0.33***
**3. Anxiety**			1	− 0.57***	− 0.37***	0.63***
**4. Peace of mind**				1	0.49***	− 0.39***
**5. Sleep quality**					1	− 0.36***
**6. Nightmare distress**						1

*Note. N* = 595; *p < *.05*, **p < *.01*, ***p < *.001*.

**Fig. 1:**
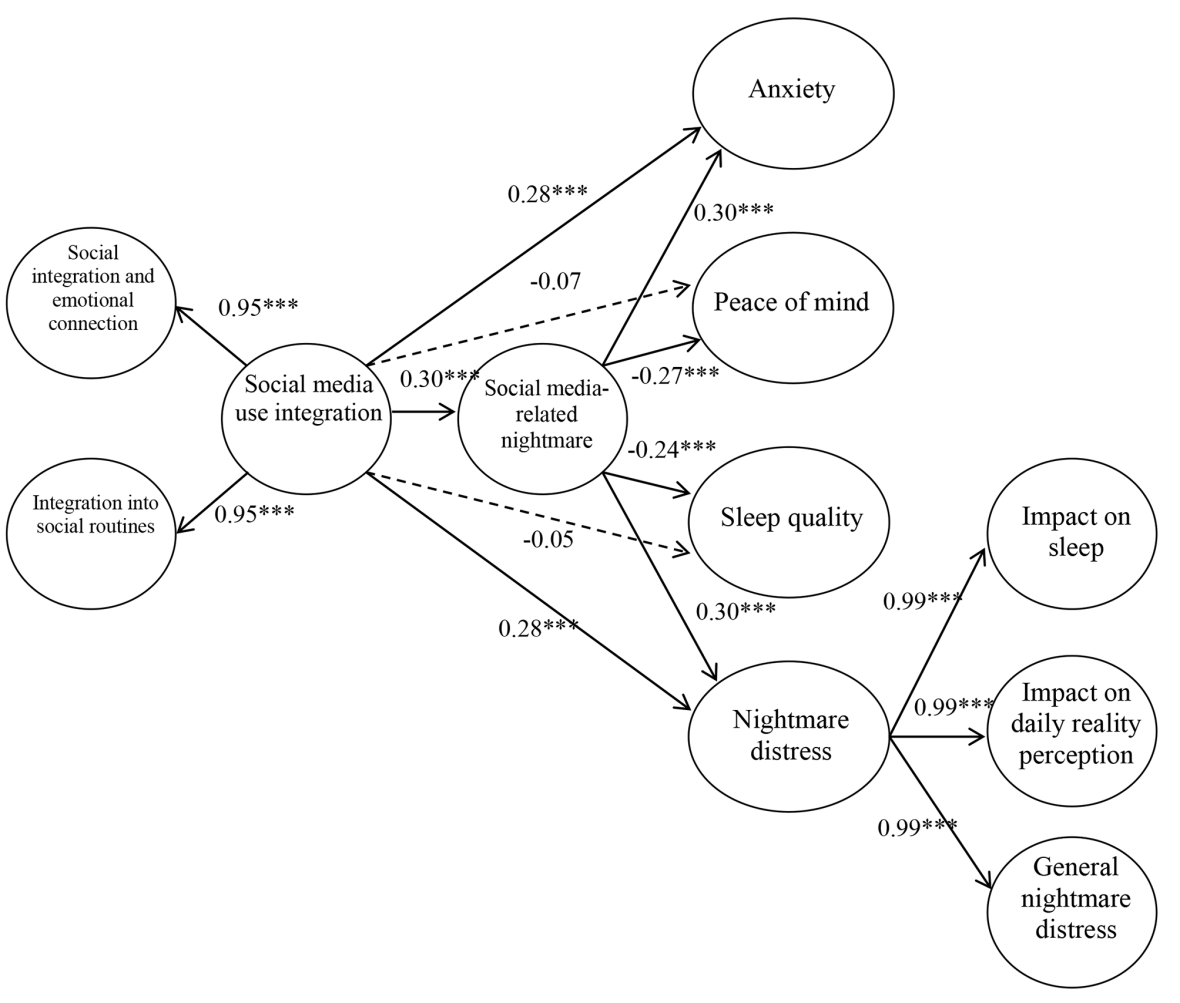
Mediation model representing the associations between social media use integration, affective well-being, and sleep difficulties, through social media-related nightmare (*N* = 595)

As a final analysis, the mediating role of social media-related nightmares was investigated in the association between social media use integration, anxiety, peace of mind, sleep quality, and nightmare distress. A second-order CFA and SEM were conducted since the Social Media Use Integration Scale (i.e., social integration/emotional connection subscale and integration into social routines subscale) and the Nightmare Distress Questionnaire (i.e., impact on sleep subscale, impact on daily reality perception subscale, and general nightmare distress subscale) had two and three subscales, respectively. The mediation model yielded good fit indices in terms of CFI, TLI, and RMSEA (*χ*^*2*^ = 1339.318, df = 708, *p* <.05;CFI = 0.951; TLI = 0.946; RMSEA = 0.039). The model is presented in Fig. 1 with standardized coefficients.

The results of the mediation analysis supported significant mediation effects of social media-related nightmares in the association of social media use integration with anxiety, peace of mind, sleep quality, and nightmare distress (see Table 5). All indirect and total effects were statistically significant (indirect effects: $\beta$ = 0.079, − 0.072, − 0.062, and 0.081 with *p* <.001, respectively; total effects: $\beta$ = 0.358 [*p* <.001], -0.137 [*p* <.01], -0.108 [*p* <.05], and 0.365 [*p* <.001], respectively). There were significant direct effects on anxiety ($\beta$ = 0.279, *p* <.001) and nightmare distress ($\beta$ = 0.284, *p*<.001), whereas no significant direct effects were observed on peace of mind and sleep quality. Overall, the positive association of social media use integration with anxiety and nightmare distress is partially mediated by social media-related nightmares. Moreover, social media-related nightmares fully mediate the association between social media use integration and peace of mind ($\beta$ = − 0.273, *p*<.001) and sleep quality ($\beta$ = − 0.235, *p*<.001) with a negative direction.

**Table 5:** Direct, indirect, and total effects from the mediation analysis

Determinant	Mediator	Outcomes	Path a	Path b	Indirect Effect	Direct Effect	Total Effect
			$\varvec{\beta }$	$\varvec{\beta }$	$\varvec{\beta }$	$\varvec{\beta }$	$\varvec{\beta }$
Social media use integration	Social media-related nightmare	Anxiety	0.265***	0.299***	0.079***	0.279***	0.358***
		Peace of mind		− 0.273***	− 0.072***	− 0.065	− 0.137**
		Sleep quality		− 0.235***	− 0.062***	− 0.046	− 0.108*
		Nightmare distress		0.304***	0.081***	0.284***	0.365***

*Note.N* = 595;*p <.05, **p <.01, ***p <.001; Path a: Determinant → Mediator; Path b: Mediator→ Outcome;$\beta$ = standardized coefficient.

## Discussion

This study aimed to quantify social media-related nightmares using the Social Media-Related Nightmare Scale (SMNS), developed for this study. The scale exhibited a unidimensional structure and demonstrated sound psychometric properties. Aligning with the continuity hypothesis of dreaming [2] and the neurocognitive model of nightmare etiology [14], the present findings suggest that the integration of social media predicts nightmares related to social media concerns and challenges.

Similar to the findings of Schred l& Göritz [4], who reported social media dreams to be quite rare, our study also revealed a low frequency of social media-related nightmares. Despite their low frequency, such nightmares were found to be negatively associated with indicators of mental health, including affective well-being and sleep quality.

Many young people are digital natives [39], born in the social media age and never knowing a time in which life was not intermingled with social media. Social media use is part of the daily life routine of many people, which can sometimes lead to addiction [40]. It appears that social media use is almost considered obligatory, to the extent that even excessive use is sometimes perceived as an adaptive form of addiction [41]. Considering the pervasive nature of social media in people’s lives and the escalating trend of excessive social media use, along with acknowledging the high emotional involvement of users with social media, investigating the potential impact of social media on sleep and dream content (especially, negative-content dreams) becomes critical. These impacts may be more noticeable for later generations due to their heightened engagement with social media.

The present results revealed that participants who integrated social media more into their lives reported a higher frequency of social media-related nightmares. While these nightmares were generally infrequent, they seemed to reflect anxieties associated with social media experiences. Drawing on the continuity hypothesis of dreaming [2] and the neurocognitive model of nightmare etiology [14], it appears that individuals spending more time on social media in their waking life are more prone to nightmares related to social media. In addition to positive or neutral manifestations of social media events and behaviors in our dreams [4], these dreams can take on a negative tone and even lead to nightmares. Users may encounter severe negative experiences, such as cyberbullying, trolling, online hate, and cyberstalking, in the social media sphere. These stressful social media events and subsequent negative affect can contribute to a high affect load and distress, which can possibly lead to nightmares. Similar to other nightmares associated with serious psychological problems (anxiety, depression, suicidal thoughts; 6), these social media-related nightmares can pose a risk to mental health.

The present findings highlighted the importance of considering psychologically adverse outcomes of social media-related nightmares, which warrant attention. These nightmares were associated with increased anxiety, lower peace of mind, poor sleep quality, and nightmare distress. These outcomes align with previous research demonstrating that excessive engagement with social media is linked to negatively valenced dreams [3, 5], poor sleep quality [17], and psychological distress [21]. This study, along with previous research, indicates that the vast and rapid adoption of social media has the potential to influence various aspects of life, including the realm of dreaming.

Furthermore, this study explored the pathway through which social media use integration is associated with sleep quality and psychological states in waking life. Findings indicated that social media-related nightmares mediate the association between the social media use integration and affective well-being as well as sleep quality. Studies have highlighted the negative impact of nightmares on quality of life and daytime function [42]. Social media-related nightmares have the potential to function as stressors, disrupting the sleep cycle of users and potentially causing awakenings during the night. Moreover, these nightmares may compromise the functionality of the sleep period, influencing processes such as the consolidation of information collected throughout the day. Consequently, the disruptions arising from social media-related nightmares could contribute to a lowered cognitive and affective well-being in waking life. This result highlights a novel pathway through which extensive social media engagement can give rise to negative content dreams about social media concerns, consequently inducing negative affective states and low-quality sleep.

The present study comes with a number of limitations. The research design was correlational, limiting our ability to establish causal relationships between the variables examined. Consequently, any interpretations about causation should be made with caution. Additionally, the present sample exclusively comprised Iranian social media users, raising questions about the generalizability of the findings to other cultural groups. Future research incorporating diverse cultural perspectives is essential for a more comprehensive understanding of the relationships observed in this study. Considering that the participants were users of different platforms (Instagram, Twitter, and Facebook), future studies focusing on social media-related nightmares within a specific platform or with the purpose of comparing social media nightmares across different platforms can provide information on whether the type of platform, such as Instagram as an image-based social media and Twitter as a text-based social media, can influence the experience of social media-related nightmares. Moreover, no information was collected on whether participants used the platforms for leisure or occupational reasons in this study. Comparing social media-related nightmares of users with different purposes of use is worth studying, as it can provide a more differentiated picture of use patterns and nightmares. Furthermore, the psychopathological profile of the participants was not assessed in this study. Given the known association of psychological disorders (e.g., anxiety disorders) with sleep quality and dream content, future investigations with controlled assessments of psychopathological states of the participants are warranted.

This study aimed to propose a novel perspective on the potential association between social media usage and mental health, sleep quality, and dreams. The concept of social media-related nightmares was introduced, and a straightforward self-report assessment was developed to quantify the frequency of these nightmares. As social media becomes increasingly intertwined with our lives, its impact extends beyond waking hours, and may even influence our dreams. The dynamic nature of social media technology continually evolves, introducing innovative platforms that hold the potential to deepen users’ dependency and integration. As this technological landscape evolves, ongoing research should persist in exploring the evolving effects of social media on users’ dreaming experiences. With the rapid advances in technology and media, including artificial intelligence (AI) and virtual reality, along with the increasing dependency on these technologies and deeper integration, it is anticipated that dreams featuring technological and media content will become more frequent. Future studies have the potential to expand the scope of this exploration, delving into areas such as nightmares related to the perceived dangers of AI.

The present study enriches existing literature by demonstrating one of the various pathways through which social media may impact affective well-being and sleep quality. The present findings indicate that increased social media use is linked with a higher frequency of nightmares centered around social media, acting as a potential conduit for anxiety and poor sleep quality. These results can provide valuable insights into the dynamic relationships between social media use, affective well-being, and sleep quality. To mitigate the occurrence of social media-related nightmares, raising awareness of sensations, thoughts, and feelings during social media consumption, and adopting a responsible and mindful use of these platforms are recommended [43].

## Data Availability

The datasets used and/or analyzed during the current study available from the first author on reasonable request.

## Declarations

### Use of Artificial Intelligence

No AI tools were used for this study.
